# The prevalence, risk factors, and outcomes of acute pulmonary embolism complicating sepsis and septic shock: a national inpatient sample analysis

**DOI:** 10.1038/s41598-024-67105-7

**Published:** 2024-07-11

**Authors:** Daisuke Hasegawa, Ryota Sato, Young Im Lee, Hong Yu Wang, Kazuki Nishida, David Steiger

**Affiliations:** 1 Department of Medicine, Mount Sinai Beth Israel, 281 1st Ave, New York, NY 10003 USA; 2 Division of Critical Care Medicine, Department of Medicine, The Queen’s Medical Center, Honolulu, HI USA; 3 Division of Pulmonary and Critical Care Medicine, Mount Sinai Beth Israel, Mount Sinai West, New York, NY USA; 4 Department of Biostatistics, Graduate School of Medicine, Nagoya University, Nagoya, Aichi Japan

**Keywords:** Sepsis, Septic shock, Pulmonary embolism, Risk factor, Outcomes, Epidemiology, Infectious-disease diagnostics

## Abstract

The study aimed to evaluate the prevalence, risk factors, and clinical outcomes of pulmonary embolism in patients diagnosed with sepsis with and without shock. The National Inpatient Sample was used to identify adults with sepsis with and without shock between 2017 and 2019. The prevalence of acute pulmonary embolism and the association of acute pulmonary embolism with in-hospital mortality, hospital length of stay for survivors, and overall costs of hospitalization were evaluated. Multivariable logistic and linear regression analyses, adjusted for various parameters, were used to explore these associations. Of the estimated 5,019,369 sepsis hospitalizations, 1.2% of patients with sepsis without shock and 2.3% of patients with septic shock developed pulmonary embolism. The odds ratio for in-hospital mortality was 1.94 (95% confidence interval (CI) 1.85–2.03, p < 0.001). The coefficient for hospital length of stay was 3.24 (95% CI 3.03–3.45, p < 0.001). The coefficient for total costs was 46,513 (95% CI 43,079–49,947, p < 0.001). The prevalence of pulmonary embolism in patients diagnosed with sepsis with and without shock was 1.2 and 2.3%, respectively. Acute pulmonary embolism was associated with higher in-hospital mortality, longer hospital length of stay for survivors, and higher overall costs of hospitalization.

## Introduction

The incidence of venous thromboembolism (VTE) in the critically ill is nearly twice that of patients admitted to the general medical floor^1^. Rates of deep venous thrombosis (DVT) in the intensive care unit (ICU) are reported to be 5–37% despite prophylaxis^2–4^. The variation in the incidence of DVT reflects the heterogeneous population in the ICU with differing rates of risk factors for VTE. Patients diagnosed with DVT in the ICU have a longer length of stay and increased mortality^4^.

Sepsis is a dysregulated inflammatory host response to infection and often progresses to multiple organ dysfunction, including coagulopathy, in 78.1% of the cases^5^. “Sepsis-associated coagulopathy (SAC)”, a systemic inflammatory response to infection, leads to coagulation activation and the formation of thrombus. SAC is defined by prolonged prothrombin time-international normalized ratio and reduced platelet count and is known to be associated with worse outcomes^6^. Also, there is an incrementally increased risk of mortality with an increase in the severity of SAC.

The surviving sepsis campaign guidelines recommend the use of pharmacologic prophylaxis to prevent VTE for adults with sepsis or septic shock as an extrapolation from prophylaxis recommendations for critically ill patients^7^. Acute pulmonary embolism (PE) can be life-threatening, and the recognition of PE risk and prevalence is extremely important. However, acute PE diagnosis in patients with sepsis may be challenging and underdiagnosed as hypotension, hypoxia, and tachycardia may be common to both sepsis with lung injury and acute PE. There is a paucity of literature describing the association between sepsis and acute PE, as well as the impact of sepsis on outcomes in patients diagnosed with acute PE. We hypothesized that the prevalence of PE was higher in the sepsis population than in the general critically ill population, and acute PE in patients hospitalized with sepsis was associated with an increased length of stay and an increase in mortality compared to patients with sepsis without acute PE.

Thus, we aimed to investigate the prevalence, risk factors, and impact on clinical outcomes of acute PE in patients with sepsis with and without shock using the national inpatient sample (NIS), a comprehensive healthcare database that includes inpatient data from all payer types.

## Methods

### Study design and data source

The NIS was queried for all adult patients (≥ 18 years) who were admitted for the primary diagnosis of sepsis from January 1st, 2017 to December 31st, 2019. Sepsis, septic shock, and PE were identified using the international classification of diseases, Tenth (ICD-10) revision codes. The NIS is the largest publicly available all-payer inpatient healthcare database designed to produce United States regional and national estimates of inpatient utilization, access, cost, quality, and outcomes. All-payer refers to the inclusion of data from all types of payers, such as Medicare, Medicaid, private insurance, uninsured patients, self-pay, no charge, or others.

Each year, the NIS collects data from over 7 million hospital stays. By applying statistical weights and adjusting for the sampling design, these data are used to evaluate more than 35 million hospitalizations nationwide. Developed through a partnership sponsored by the Agency for Healthcare Research and Quality and the Healthcare Cost and Utilization Project, the NIS helps inform decision-making at national, state, and community levels.

### Study population

We identified sepsis with the primary diagnosis of any of the ICD-10 codes specified in the denominator for the Centers for Medicare and Medicaid Services SEP-1 measure, including septicemia, sepsis, severe sepsis, and septic shock^8^. Septic shock was defined as the primary diagnosis of “septic shock”, the primary diagnosis of “sepsis” and the secondary diagnosis of “septic shock”, or the primary diagnosis of “sepsis” and the procedure codes of “vasopressor support”^9^. PE was defined using the diagnosis codes previously validated^10^. Details of these codes are shown in Supplemental Table 1. Inclusion criteria were: (1) age ≥ 18 years and (2) sepsis. Ages over 89 are aggregated into a single category of 90 years old in this dataset.

### Outcomes

We described the prevalence of acute PE among patients with sepsis with and without shock. We also investigated the risk factors for developing acute PE, the association between the presence of acute PE and in-hospital mortality, hospital length of stay among survivors, and total hospital charges incurred in patients with sepsis.

### Statistical analysis

We provided the mean and standard error of the mean for continuous variables and percentages for categorical variables. To explore the risk factors for the development of acute PE, we performed a multivariable logistic regression analysis with explanatory variables, including categorical variables (septic shock, sex, weekend admission, race, primary payers, hospital characteristics such as bed size, location/teaching status, region, and median household income national quartile for the patient ZIP code) and continuous variables (age, year of the data [2017, 2018, or 2019], Elixhauser comorbidity index). To investigate the association between acute PE and the outcome after adjusting for confounding factors, we used multivariable logistic regression and linear regression analyses: (a) acute PE and in-hospital mortality using logistic regression, (b) acute PE and hospital length of stay (days) among survivors using linear regression, and (c) acute PE and total hospital charges (dollar) using linear regression. In each case, we adjusted for categorical variables (sex, weekend admission, race, primary payers, hospital characteristics such as bed size of hospital, location/teaching status of hospital, region of hospital, and median household income national quartile for the patient ZIP code) and continuous variables (age, year of the data [2017, 2018, or 2019], Elixhauser comorbidity index). We calculated the variance inflation factor for each variable for both the analysis of risk factors and mortality analysis to ensure that there was no significant multicollinearity in the models. We conducted an additional analysis to examine the association between PE and mortality after adjusting for septic shock. Septic shock was included as an additional variable in our existing multivariable logistic regression model, using the same methodology described previously to assess the impact of septic shock on the association between PE and mortality. Age, median incomes of the hospital area, primary payers, race, sex, mortality, and weekend admission were missing in 0.0016, 1.9, 0.11, 2.3, 0.0047, and 0.0004%, respectively. We performed multiple imputations using chained equations, repeating the process 20 times with different random seeds for the missing values. The remaining variables contained no missing data across the entire population. All statistical analyses were performed using Stata MP version 17 (StataCorp, College Station, TX). Statistical significance was defined as P < 0.05. The institutional review board of the Mount Sinai Health System approved this study (STUDY-22-01281).

### Ethics approval and consent to participate

Local IRB exempted (#STUDY-22-01281).

## Results

A total of 1,003,874 hospitalizations with the primary diagnosis of sepsis were identified (sepsis without shock: 810,165 hospitalizations, septic shock 193,709 hospitalizations). The national estimate of hospitalizations for sepsis was 5,019,369 hospitalizations (sepsis without shock: 4,050,824 hospitalizations, septic shock: 968,545). While 1.2% (49,200/4,050824) had an acute PE in patients with sepsis without shock, 2.3% (21,815/968,545) had an acute PE in patients with septic shock. The details regarding the characteristics of hospitalizations are shown in Table 1.

**Table 1:** Patient’s characteristics.

	Sepsis (n = 1,003,874) national estimate (n = 5,019,369)	
	Acute pulmonary embolism (+) national estimate (n = 71,015)	Acute pulmonary embolism (−) national estimate (n = 4,948,354)
Age, year (mean [SEM])	64.5 (0.05)	66.9 (0.13)
Female (%)	50.40%	50.40%
Elixhauser comorbidity index (mean [SEM])	4.2 (0.01)	5.7 (0.02)
Charlson comorbidity index (mean [SEM])	3.2 (0.02)	2.7 (0.01)
Septic shock (%)	30.70%	19.10%
Bed size of hospital (%)		
1. Small	1. 18.6%	1. 22.1%
2. Medium	2. 30.1%	2. 31.1%
3. Large	3. 51.2%	3. 46.8%
Location/teaching status of hospital (%)		
1. Rural	1. 8.4%	1. 10.0%
2. Urban nonteaching	2. 19.8%	2. 24.1%
3. Urban teaching	3. 71.8%	3. 65.9%
Region of hospital (%)		
1. Northeast	1. 16.1%	1. 16.1%
2. Midwest	2. 21.1%	2. 20.3%
3. South	3. 39.2%	3. 39.8%
4. West	4. 23.6%	4. 23.8%
Primary expected payer (%)		
1. Medicare	1. 58.4%	1. 61.2%
2. Medicaid	2. 16.4%	2. 14.1%
3. Private insurance	3. 17.6%	3. 16.4%
4. Self-pay	4. 3.9%	4. 4.0%
5. No charge	5. 4.5%	5. 3.5%
6. Other	6. 2.3%	6. 2.1%
Race (%)		
1. White	1. 70.9%	1. 69.3%
2. Black	2. 16.3%	2. 13.3%
3. Hispanic	3. 7.7%	3. 11.0%
4. Asian/Pacific Islander	4. 2.1%	4. 3.0%
5. Native American	5. 0.6%	5. 0.8%
6. Other	6. 2.5%	6. 2.7%
Year (%)		
1. 2017	1. 30.8%	1. 32.2%
2. 2018	2. 34.3%	2. 33.7%
3. 2019	3. 34.9%	3. 34.0%
Median household income national quartile for patient ZIP code (%)		
1. Fisrt quartile	1. 31.7%	1. 31.3%
2. Second quartile	2. 26.3%	2. 26.6%
3. Third quartile	3. 23.6%	3. 23.6%
4. Fourth quartile	4. 18.4%	4. 18.5%
Weekend admission (%)	26.60%	26.80%

*SEM* standard error of the mean, *CS* cardiogenic shock, *IQR* interquartile range, *OR* odds ratio, *CI* confidence interval, *ZIP* zone improvement plan.

In the multivariable logistic regression model, statistically significant risk factors included septic shock, younger age, higher elixhauser comorbidity index, medium or large hospitals, urban teaching hospitals, White or Black race, and higher median household income of patient’s ZIP code. Details regarding factors associated with developing acute PE are shown in Table 2.

**Table 2:** Risk factors for acute pulmonary embolism.

Variables	OR (95% CI) for acute pulmonary embolism	P-value
Septic shock	1.40 (1.35–1.46)	< 0.001
Age	0.99 (0.99–0.99)	< 0.001
Female	1.02 (0.98 –1.05)	0.356
Weekend admission	1.01 (0.97–1.05)	0.685
Elixhauser comorbidity index	1.18 (1.17–1.19)	< 0.001
Bed size of hospital		
1. Small	1.00	Reference
2. Medium	1.12 (1.06–1.18)	< 0.001
3. Large	1.24 (1.18–1.31)	< 0.001
Location/teaching status of hospital		
1. Rural	1.00	Reference
2. Urban nonteaching	0.92 (0.86–0.99)	0.044
3. Urban teaching	1.17 (1.09–1.26)	< 0.001
Region of hospital		
1. Northeast	1.00	Reference
2. Midwest	0.88 (0.82–0.93)	< 0.001
3. South	0.96 (0.91–1.02)	0.172
4. West	0.99 (0.93–1.05)	0.754
Primary expected payer		
1. Medicare	1.00	Reference
2. Medicaid	1.29 (1.22–1.36)	< 0.001
3. Private insurance	1.31 (1.25–1.38)	< 0.001
4. Self-pay	1.41 (1.29 – 1.56)	< 0.001
5. No charge	1.90 (1.50–2.42)	< 0.001
6.Other	1.30 (1.16–1.45)	< 0.001
Race		
1. White	1.00	Reference
2. Black	1.04 (0.99–1.09)	0.156
3. Hispanic	0.67 (0.62–0.71)	< 0.001
4. Asian/Pacific Islander	0.65 (0.58–0.74)	< 0.001
5. Native American	0.63 (0.50–0.79)	< 0.001
6. Other	0.88 (0.79–0.99)	0.031
Year		
1. 2017	1.00	Reference
2. 2018	1.04 (0.99–1.10)	0.087
3. 2019	1.04 (0.99–1.09)	0.149
Median household income national quartile for patient’s ZIP code		
1. Fisrt quartile	1.00	Reference
2. Second quartile	1.03 (0.98–1.07)	0.284
3. Third quartile	1.04 (0.99–1.10)	0.086
4. Fourth quartile	1.06 (1.00–1.12)	0.037

In the multivariable logistic and linear regression models, the presence of acute PE was associated with significantly higher odds for in-hospital mortality (adjusted odds ratio: 1.94, 95% confidence interval [CI] 1.85–2.03, p < 0.001), longer hospital length of stay (coefficient: 3.24, 95% CI 3.03–3.45, p < 0.001), and higher total costs (coefficient: 46,513, 95% CI 43,079–49,947, p < 0.001).

The variance inflation factor values were sufficiently low, indicating no significant multicollinearity for risk factors and mortality analysis (Supplemental Table 2).

The additional analysis showed that after adjusting for septic shock, the odds ratio for the association between PE and mortality was 1.81 (95% CI 1.72–1.90) with a p-value < 0.001.

## Discussion

In the present study, we reported that the prevalence of acute PE among patients with sepsis without shock was 1.2%, whereas the prevalence of PE in patients with septic shock was 2.3%. In hospitalized patients with sepsis, acute PE was associated with higher in-hospital mortality, a longer length of hospital stay among survivors, and higher total hospital costs. This study also revealed the influence of possible socioeconomic disparities associated with hospital size, hospital location, hospital teaching type, patient insurance status, patient income, and risk of acute PE in patients with sepsis with and without shock.

This is the first study to report the prevalence of acute PE in patients with both sepsis with and without shock, the impact of PE on clinically relevant outcomes in patients with sepsis, and the potential risk factors for PE in this patient cohort. Although septic patients may develop a “sepsis-associated coagulopathy”^11–16^, this study demonstrated that the prevalence of acute PE in sepsis was not significantly higher than the described rate of 2–4% in the critically ill patient population^7^.

This study identified demographic and clinical factors associated with the development of acute PE. We demonstrate that younger age, increased comorbidities as conveyed in the elixhauser comorbidity index, and white or black race were associated with an increased risk of developing acute PE, and there was a decreased rate in Hispanic and Asian/Pacific Islanders. For age and risk of PE, the incidence of VTE increases with age and is eight times higher at ages greater than 80 years than in the fifth decade of life^17^. A significant number of factors predispose to VTE, including increased age, genetic and acquired risk factors. Still, ultimately, VTE occurs due to a concurrence of preexisting risk factors and often temporary, acute risk factors for VTE, such as sepsis^18^. For our finding that younger age was a risk factor for PE, the causes are probably multifactorial. One possible significant reason is the use of ICD-10 codes, which capture only definite diagnoses of PE rather than possible PE and the threshold for performing diagnostic imaging might be lower in younger patients due to their better overall prognosis as compared to the older patients.

Race—Black Americans have decreased access to health care, and experience more severe morbidity associated with many chronic illnesses^19,20^. Black patients are almost twice as likely to be hospitalized for PE and have a 50% higher age-standardized PE-related death rate compared to white patients, underscoring the complexities associated with the incidence and outcomes of PE in Black patients^21,22^. In contrast, the incidence of VTE is reported to be much lower in Hispanic and Asian/Pacific Islanders compared to whites or blacks^23–25^. Asian/Pacific Islanders have a lower prevalence of both idiopathic and provoked thromboembolic disease. Hispanics have a significantly lower prevalence of VTE compared to Caucasians, but the prevalence is greater than in Asian/Pacific Islanders^26,27^. The role of ethnicity and risk of PE may be further explained by analyzing the incidence of thrombophilic disorders in different ethnic groups. Factor V Leiden and the prothrombin mutation are more frequent in white Caucasians than Blacks or East Asians; however, Blacks have higher levels of Factor VIII, Von Willebrand Factor, plasmin-antiplasmin complex, and D-dimer. The clinical significance of these factors in acute sepsis is unclear^27^.

Our study identified non-biological factors associated with an increased risk of developing acute PE that included an increased size of the hospital, urban teaching hospitals, hospitals located in the northeast of the US, self-payer and “no charge” status coverage, and an increase in median household income national quartile for the patient’s ZIP code. Urban teaching hospitals have traditionally managed socioeconomically deprived patients and the uninsured. Neighborhood socioeconomic disadvantage can be expressed in the “area deprivation index,” a validated publicly available index established from US Census data^28^. Area deprivation is associated with inequalities in US mortality secondary to slower declines in mortality in more deprived areas^29–31^. Paradoxically, we determined that there was an increase in PE in patients with higher median household income as deduced by the national quartile for the patient’s ZIP code. This is unexpected if we believe, as described above, that the risk of infection, and incidence of PE is higher in patients with socioeconomic deprivation. While ZIP codes are used in large population studies to define socioeconomic status, ZIP codes often disguise the significant heterogeneity in socio-economic status of the studied population compared to the use of different area-based metrics, such as the use of census block data^32^. Lastly, it has been demonstrated that higher socioeconomic status is associated with higher utilization of diagnostic imaging studies that may be associated with increases in the diagnosis of PE^33^. The higher incidence of PE in patients with septic shock than sepsis without shock is possibly due to the greater degree of inflammation in patients with septic shock, where greater inflammation plays a major role in sepsis-associated coagulopathy^11^.

There are certain limitations to the study. We relied on the ICD-10 codes for the diagnosis of sepsis, septic shock, and PE, which have been previously used or validated in prior studies, but it is possible that there were variations in the accurate use of diagnostic criteria to define sepsis and septic shock^34^. In addition, it is possible that the reported incidence of PE could have been influenced by differing thresholds for working up possible PE between hospitals. Moreover, since this is a study using big data, individual-level data were not available, and thus, hidden confounders may have been responsible for the observed results.

In conclusion, we determined that the prevalence of PE in patients diagnosed with sepsis with and without shock was 1.2 and 2.3%, respectively. Acute PE was associated with higher in-hospital mortality, longer hospital length of stay for survivors, and higher overall costs of hospitalization. A prospective study is warranted where the incidence of PE is evaluated in patients with sepsis and septic shock and where there is a standardized management protocol for evaluating and treating PE.

### Supplementary Information

Supplementary Table 1.

Supplementary Table 2.

## Data Availability

The datasets used and analyzed during the current study are available from the corresponding author upon reasonable request.
