# Supplementary material for: The prevalence, risk factors, and outcomes of acute pulmonary embolism complicating sepsis and septic shock: a national inpatient sample analysis
Source: Sci Rep. 2024 Jul 11;14:16049. doi: 10.1038/s41598-024-67105-7 (PMC11239923; doi:10.1038/s41598-024-67105-7)
Supplement: Supplementary file 1 — Supplementary Table 1. [file 41598_2024_67105_MOESM1_ESM.docx]

**Supplemental Table 1: ICD-10 codes for the diagnosis of Sepsis, Septic shock and Pulmonary Embolism**

**ICD-10 codes for Sepsis**

A02.1: Salmonella sepsis

A22.7: Anthrax sepsis

A26.7: Erysipelothrix sepsis

A32.7: Listerial sepsis

A40.0 Sepsis due to streptococcus, group A

A40.1: Sepsis due to streptococcus, group B

A40.3: Sepsis due to Streptococcus pneumoniae

A40.8: Other streptococcal sepsis

A40.9: Streptococcal sepsis, unspecified

A41.01: Sepsis due to Methicillin susceptible Staphylococcus aureus

A41.02: Sepsis due to Methicillin resistant Staphylococcus aureus

A41.1: Sepsis due to other specified staphylococcus

A41.2: Sepsis due to unspecified staphylococcus

A41.3: Sepsis due to Hemophilus influenzae

A41.4: Sepsis due to anaerobes

A41.50: Gram-negative sepsis, unspecified

A41.51: Sepsis due to Escherichia coli

A41.52: Sepsis due to Pseudomonas

A41.53: Sepsis due to Serratia

A41.59: Other Gram-negative sepsis

A41.81: Sepsis due to Enterococcus

A41.89: Other specified sepsis

A41.9: Sepsis, unspecified organism

A42.7: Actinomycotic sepsis

A54.86: Gonococcal sepsis

B37.7: Candidal sepsis

R65.20: Severe sepsis without septic shock

R65.21: Severe sepsis with septic shock

**ICD-10-PS code for Vasopressor use**

3E043XZ: Introduction of Vasopressor into Central Vein, Percutaneous Approach

3E033XZ: Introduction of Vasopressor into Peripheral Vein, Percutaneous Approach

**ICD-10 code for Pulmonary Embolism**

I26.01: Septic pulmonary embolism with acute cor pulmonale

I26.02: Saddle embolus of pulmonary artery with acute cor pulmonale

I26.09: Other pulmonary embolism with acute cor pulmonale

I26.90: Septic pulmonary embolism without acute cor pulmonale

I26.92: Saddle embolus of pulmonary artery without acute cor pulmonale

I26.99: Other pulmonary embolism without acute cor pulmonale
