# Supplementary material for: The prevalence, risk factors, and outcomes of acute pulmonary embolism complicating sepsis and septic shock: a national inpatient sample analysis
Source: Sci Rep. 2024 Jul 11;14:16049. doi: 10.1038/s41598-024-67105-7 (PMC11239923; doi:10.1038/s41598-024-67105-7)
Supplement: Supplementary file 2 — Supplementary Table 2. [file 41598_2024_67105_MOESM2_ESM.docx]

**Supplemental Table 2: Variance Inflation Factor (VIF) for Mortality and Risk Factor Analyses**

1. **Risk Factor**

| **Variables** | **VIF** |
| --- | --- |
| Septic shock | 1.05 |
| Female | 1.00 |
| Elixhauser Comorbidity Index | 1.19 |
| Age | 1.78 |
| Bed size of hospital  1.Small  2.Medium  3.Large | 1.00  1.68  1.72 |
| Location/teaching status of hospital  1.Rural  2.Urban nonteaching  3.Urban teaching | 1.00  2.84  2.93 |
| Region of hospital  1. Northeast  2. Midwest  3. South  4. West | 1.00  1.86  2.25  1.97 |
| Primary expected payer  1.Medicare  2.Medicaid  3.Private insurance  4.Self-pay  5.No charge  6.Other | 1.00  1.53  1.38  1.21  1.02  1.04 |
| Race  1.White  2.Black  3.Hispanic  4.Asian/Pacific Islander  5.Native American  6.Other | 1.00  1.11  1.13  1.06  1.01  1.02 |
| Year  1.2017  2.2018  3.2019 | 1.00  1.36  1.37 |
| Median household income national quartile for patient ZIP Code  1.Fisrt quartile  2.Second quartile  3.Third quartile  4.Fourth quartile | 1.00  1.41  1.48  1.53 |
| Weekend admission | 1.00 |

1. **Mortality**

| **Variables** | **VIF** |
| --- | --- |
| Pulmonary embolism | 1.01 |
| Female | 1.00 |
| Elixhauser Comorbidity Index | 1.15 |
| Age | 1.79 |
| Bed size of hospital  1.Small  2.Medium  3.Large | 1.00  1.68  1.72 |
| Location/teaching status of hospital  1.Rural  2.Urban nonteaching  3.Urban teaching | 1.00  2.84  2.92 |
| Region of hospital  1. Northeast  2. Midwest  3. South  4. West | 1.00  1.86  2.25  1.97 |
| Primary expected payer  1.Medicare  2.Medicaid  3.Private insurance  4.Self-pay  5.No charge  6.Other | 1.00  1.53  1.38  1.21  1.02  1.04 |
| Race  1.White  2.Black  3.Hispanic  4.Asian/Pacific Islander  5.Native American  6.Other | 1.00  1.11  1.13  1.06  1.01  1.02 |
| Year  1.2017  2.2018  3.2019 | 1.00  1.36  1.37 |
| Median household income national quartile for patient ZIP Code  1.Fisrt quartile  2.Second quartile  3.Third quartile  4.Fourth quartile | 1.00  1.41  1.48  1.53 |
| Weekend admission | 1.00 |
